# Supplementary material for: The interactome of intact mitochondria by cross-linking mass spectrometry provides evidence for coexisting respiratory supercomplexes
Source: Mol Cell Proteomics. 2017 Dec 8;17(2):216–32. doi: 10.1074/mcp.RA117.000470 (PMC5795388; doi:10.1074/mcp.RA117.000470)
Supplement: Supplemental Data [file supp_RA117.000470_133922_0_supp_23978_fzffwf.zip › spectra_annotation/mito_DR_spectra_annotation/21-1-7-1-2-1.pdf]

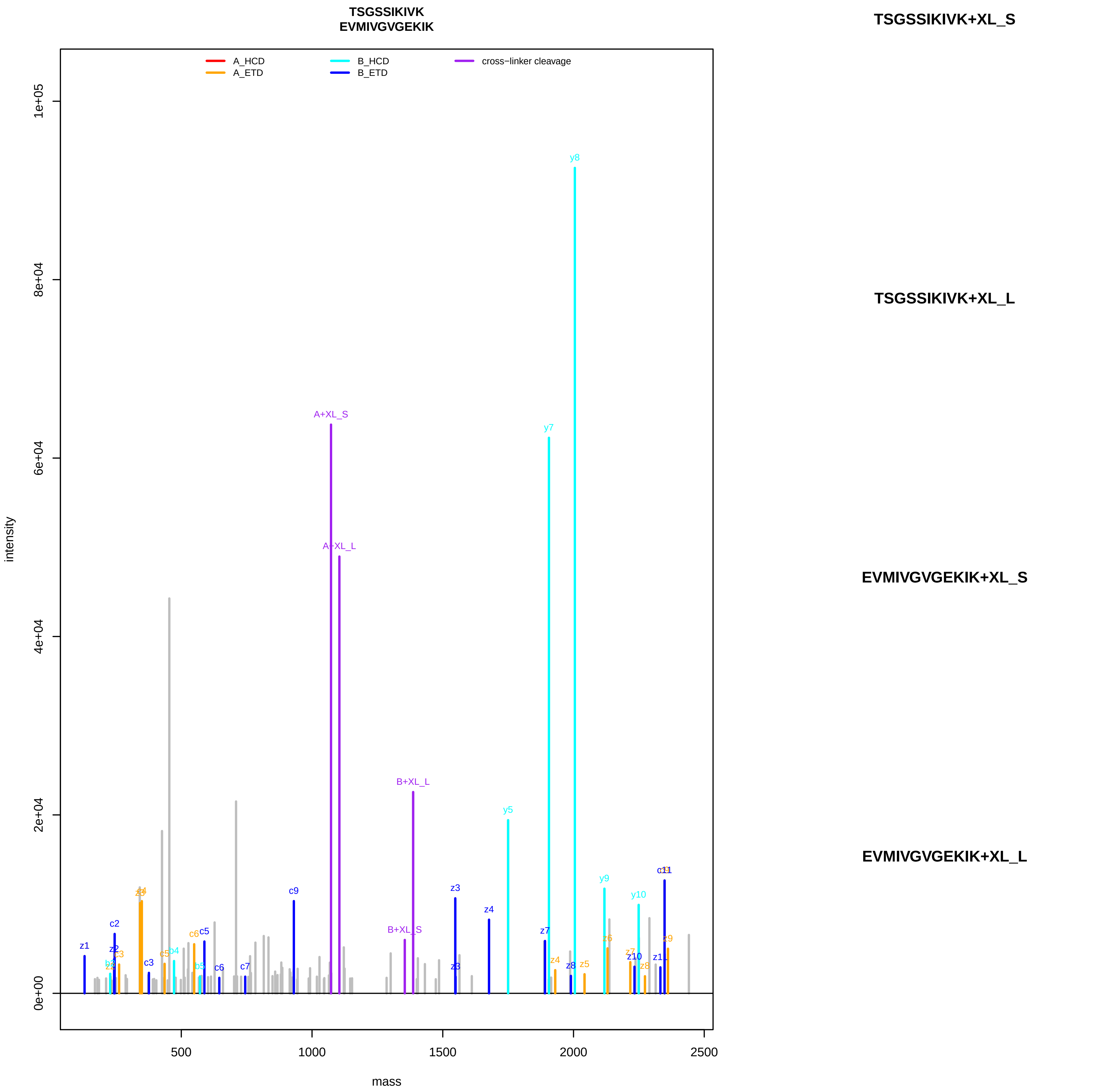
TSGSSIKIVK
EVMIVGVGEKIK
A_HCD
A_ETD
B_HCD
B_ETD
cross−linker cleavage
intensity
mass
A+XL_S
A+XL_L
B+XL_L
B+XL_S
y8
y7
y5
y9
y10
c9
z3
z4
z7
c2
c5
c6
z1
TSGSSIKIVK+XL_S
TSGSSIKIVK+XL_L
EVMIVGVGEKIK+XL_S
EVMIVGVGEKIK+XL_L
